# Increased incidence of blood culture contaminations during and after the COVID-19 pandemic

**DOI:** 10.1007/s15010-024-02469-6

**Published:** 2025-03-03

**Authors:** Hannah Tolle, Aude Nguyen, Aleece MacPhail, Nasreen Hassoun-Kheir, Marie-Noelle Chraiti, Filippo Boroli, Marie-Céline Zanella, Stephan Harbarth, Gaud Catho, Niccolò Buetti

**Affiliations:** 1 Infection Control Program and World Health Organization Collaborating Centre, Faculty of Medicine, Geneva University Hospitals, Geneva, Switzerland; 2 Department of Medicine I, Gastroenterology and Hepatology, University Hospital Carl-Gustav-Carus, Technische Universität Dresden (TU Dresden), Dresden, Germany; 3 Department of Infectious Diseases, Monash Health, Melbourne, Australia; 4 School of Public Health and Preventive Medicine, Monash University, Melbourne, Australia; 5 Division of Intensive Care, Geneva University Hospitals, Geneva, Switzerland; 6 Division of Infectious Diseases, Central Institute, Valais Hospital, Sion, Switzerland; 7 INSERM, IAME, Université Paris-Cité, Paris, 75006 France

**Keywords:** Blood culture, Contamination, COVID-19 pandemic

## Abstract

**Purpose:**

Blood culture contamination (BCC) is mainly caused by commensal bacteria, during sample collection. It results in unnecessary antibiotic exposure, prolonged hospitalisation, additional microbiology workup and significant adverse health-economic burden. We aimed to investigate the short- and long-term impact of the COVID-19 pandemic on the incidence of BCC.

**Methods:**

We conducted a retrospective, observational cohort study at Geneva University Hospitals (HUG). We included all BCCs from January 2018 to December 2023, collected as part of a prospective hospital-wide surveillance by the infection control team. Data were analyzed using segmented Poisson regression models to evaluate BCC incidence rate ratios (IRRs) across three periods: pre-COVID-19 (2018–2019), during COVID-19 (2020–2021), and post-COVID-19 peak (2022–2023).

**Results:**

Out of 456,873 collected blood cultures, 1,247 BCCs were identified (0.27%). The contamination rate per 1000 samples increased from 1.53 pre-COVID-19 to 2.94 during COVID-19 and 3.52 post-COVID-19. Compared to the pre-COVID-period, incidence rate ratios (IRRs) for BCC increased during COVID-19 (IRR 1.84, 95% CI 1.58–2.15) and post-COVID-19 peak (IRR 2.29, 95% CI 1.97–2.66). During COVID-19, proportions of BCC were increased in intensive care units (27.4%, *n* = 127) and returned to baseline level post-COVID-19 (17.3%, *n* = 93, *p* < 0.001); whereas, in other wards, BCC remained elevated (42.2%, *n* = 227) in the post-COVID-19 period.

**Conclusions:**

We observed a significant rise in BCC incidence during and after the COVID-19 peak. The persistently elevated post-peak rates highlight ongoing challenges in regaining optimal aseptic blood culture collection practices and the need for further exploration of persisting factors increasing BCC rates.

**Supplementary Information:**

The online version contains supplementary material available at 10.1007/s15010-024-02469-6.

## Introduction

Blood cultures are a key diagnostic tool for clinicians, and a sensitive method for identifying bloodstream infections. By determining the causative organisms and their antimicrobial susceptibilities, clinicians obtain needed information to provide adequate treatment and improve patient outcome. However, blood culture contamination (BCC) occurs frequently, and represents a persisting problem in modern medical care [1]. Reported BCC incidence varies from 0.6 to 17% of collected blood cultures depending on the settings [2].

A contaminant is defined as a microorganism that is introduced into the sample during specimen collection or processing, and that is therefore not pathogenic to the patient. The most frequent contaminants are commensal skin flora such as coagulase-negative staphylococci (CoNS), *Bacillus* spp., viridans group streptococci, *Corynebacterium* spp. and *Cutibacterium* spp.

Differentiating contamination from true bacteremia is challenging, since these microorganisms can also cause invasive infections, leading to diagnostic uncertainty [3]. There is also a lag between preliminary and definitive results of blood culture, during which it may not be clear whether the isolated organism is a commensal skin organism or a more virulent pathogen such as *Staphylococcus aureus*, which requires prompt treatment. Therefore, BCC has significant clinical and economic consequences, including administration of inappropriate antibiotic treatments, diagnostic bias and delay, unnecessary insertion or replacement of invasive equipment, and additional investigations [4]. The estimated economic burden ranges between $2,923 and $5,812 for total additional hospital costs attributable to a false-positive blood culture per patient [5].

An increased BCC incidence during the COVID-19 pandemic was observed in several studies [6]. Yet, long-term evidence on the impact of the pandemic on BCC incidence is lacking. The primary objective of this study was to report the incidence of BCCs in a large hospital network derived from prospectively collected hospital surveillance data from 2018 to 2023, and to compare incidence in the pre- during- and post-COVID-19 peak periods.

## Methods

### Setting, study design and data sources

We performed a large observational study on BCCs rates based on surveillance data from Geneva University Hospitals (HUG). HUG is Switzerland’s largest tertiary care center network, with 2’100 beds and approximately 60′000 hospital admissions per year. It includes acute-care, geriatrics, pediatrics, gynecology-obstetrics and psychiatry services as well as five rehabilitation and/or palliative care sites. We analyzed all BCC episodes observed at the whole HUG hospital network between 1st January 2018 and 31st December 2023. The data used in this study was collected prospectively within the framework of regular infection prevention and control (IPC) surveillance at HUG. The total number of blood culture samples collected annually were extracted from the laboratory registry data.

### Definitions

A blood culture is defined as a blood sample submitted for culture from a single blood draw, consisting of two bottles set (aerobic and anaerobic). Each individual bottle is counted in the total number of samples collected; the total number of samples collected equals the total number of bottles collected. Positive blood cultures were prospectively classified by the same team of trained medical staff (IPC nurses) throughout the study period in four distinct categories, namely BCC, secondary bloodstream infection, catheter related bloodstream infection and other primary bloodstream infection according to ECDC criteria [7]. Briefly, skin commensals and bacteria of the *Streptococcus viridans* group were not considered contaminants if they were identified at least in two blood culture sets at different times (except in the neonatology ward, where, according to ECDC criteria, single positive blood culture was considered a true bacteremia) and in the presence of clinical symptoms and signs of infection (i.e., temperature *> 38*^*0*^*C*,* chills or hypotension).* CoNS (except *S. lugdunensis*), *Bacillus spp*,* Cutibacterium* spp, *Corynebacterium* spp, and *Micrococcus* spp were considered typical skin commensals. In 2021, the referral list of skin commensals has been slightly enlarged to the referral list of common commensals according to the CDC/NHSN definitions. Further details are provided in the supplementary methods.

Blood cultures were incubated using BACTEC™ (Becton, Dickinson Microbiology Systems) for a minimum of 5 days. Pathogen identification and antimicrobial susceptibility testing were conducted by MALDI-TOF/MS, AST by disk diffusion and interpreted according to the European Committee on Antimicrobial Susceptibility Testing (EUCAST, v05-v11).

The first cases of COVID-19 occurred in Switzerland in February 2020. For our study purposes, pre-COVID-19 period was defined as the period from 2018 until 31st December 2019 for simplicity. We defined the post-COVID-19 period after the pandemic peak in 2020/2021, starting with the circulation of Omicron variant (which had a less severe disease course and therefore reduced impact on healthcare system), from January 2022. The resulting periods, based on impact of COVID-19 on the healthcare system, were: pre-COVID-19 (2018–2019), during-COVID-19 (2020–2021) and post-COVID-19 peak (2022–2023), lasting 2 years each. BCC prevention measures, implemented at HUG are reported in the supplementary material.

### Statistical analyses

For statistical analysis, we used the total blood culture samples drawn as denominator. First, we compared BCCs across our three periods of interest (pre-COVID-19, during COVID-19 and post-COVID-19 peak) according to several prespecified groups (hospital departments, adults *versus* pediatric, type of microorganism), using Chi-square, Fisher or Kruskal-Wallis test. Second, we graphically described the monthly incidence rates of BCCs per 1,000 blood culture samples drawn, stratified by different features. The absolute monthly number of BCCs stratified by three periods of interest was illustrated graphically for the study period. Third, we evaluated incidence rate ratios (IRRs) for BCCs by segmented Poisson regression models using aggregated monthly data; with the pre-COVID-19 period as the referent and number of blood cultures drawn as the offset. Segmented Poisson regression analysis is commonly used in infection control studies to analyze changes in the incidence of infections over time and to evaluate the impact of interventions. This approach is particularly suited for count data (e.g., infection counts) and can account for time-dependent changes.

We tested for overdispersion by using the likelihood ratio test and used a negative binomial model, if required. We performed a sensitivity analysis that excluded the microorganisms that were introduced in 2021. We also conducted another sensitivity analysis assessing different period stratification (i.e., pre-COVID-19, early COVID-19 (2020), late COVID-19 (2021) and post-COVID-19 periods). We used SAS version 9.4 (SAS Institute, Inc., https://www.sas.com) and R (version 4.3.3) to perform all analyses and considered *p* < 0.05 statistically significant. We report our findings according to the STROBE guidelines. The analysis was performed on anonymized non-genetic surveillance data as part of routine quality activities. Ethical consent was not required according to Swiss law (Article 33, Paragraph 2, Human Research Act).

## Results

During the study period, a total of 456’873 blood culture bottles were drawn at HUG and 1247 BCCs were observed (0,27%). The median age of patients with BCCs was 62 years (interquartile range [IQR] 39–76); and 742 (59.5%) were male.

### Characteristics of BCC during the three periods

The number of BCCs observed in the pre-, during- and post-COVID-19 period differed, and were 252, 464 and 531, respectively (Table 1).

**Table 1:** Contamination characteristics in the pre-, during-, and post-COVID-19-peak period

	Pre-COVID-19 *n* (%)	During-COVID-19 *n* (%)	Post-COVID-19 *n* (%)	*p*-value
Total contaminations	252	464	531	
Sex (*%*)				0.003
Male	144 (*57.1*)	307 (*66.2*)	291 (*54.8*)	
Median Age (IQR) in years	59.5 (31.5-72.25)	61.5 (42.0–77.0)	63 (39.0–77.0)	0.061
Settings (*%*)				< 0.001
Adult Intensive Care units	44 (*17.5*)	127 (*27.4*)	93 (*17.3*)	
Regular Ward	111 (*44.1*)	168 (*36.3*)	227 (*42.2*)	
Emergency	95 (*37.7*)	168 (*36.3*)	198 (*36.8*)	
Outpatient department	2 (*0.8*)	1 (*0.2*)	13 (*2.4*)	
Patient Group (*%*)				0.0035
Adult	197 (*78.2*)	407 (*87.7*)	449 (*84.5*)	
Paediatric	55 (*21.8*)	57 (*12.3*)	82 (*15.4*)	
Pathogen Group (*%*)				0.017
CoNS	186 (*73.8*)	315 (*67.9*)	351 (*66.1*)	
*Streptococci*	4 (*1.6*)	17 (*3.7*)	34 (*6.4*)	
Anaerobes	16 (*6.4*)	19 (*4.1*)	24 (*4.5*)	
Polymicrobial	21 (*8.3*)	41 (*8.8*)	46 (*8.7*)	
Other^1^	25 (*9.9*)	72 (*15.5*)	76 (*14.3*)	
Total samples collected	164 868	157 328	152 677	
Incidence rate ratio (95% CI)	(reference)	1.84 (1.58–2.15)	2.29 (1.97–2.66)	*p* < 0.001
Contamination rate/1000 samples	1.53	2.94	3.52	
Contamination incidence/ 10 000 patient days	1.68	3.59	3.83	
Patient days	1 496 396	1 293 388	1 384 321	

Legend. CoNS: Coagulase-negative Staphylococci, IQR: Interquartile Range

1 Other included: *Corynebacterium* spp, *Bacillus* and *Micrococcus*

During-COVID-19, an increased proportion of contaminations occurred in the intensive care units (*n* = 127, 27.43%) compared to pre- (*n* = 44, 17.46%), and post-COVID-19 periods (*n* = 93, 17.29%). The proportion of BCCs in the emergency department remained stable during COVID-19, and a minor proportion occurred in regular hospital wards (*n* = 168, 36.3%) compared to the pre- (*n* = 111, 44.1%) and post-COVID-19 period (*n* = 227;42.2%).

The most frequently identified pathogens were CoNS (*n* = 852, 67.9%), anaerobes (*n* = 59, 5.0%) and polymicrobial (*n* = 108, 8.6%). The proportion of CoNS decreased during the study period, while the proportion of *Streptococci* slightly increased (supplementary Table 1).

### Temporal trends

**Fig. 1:**
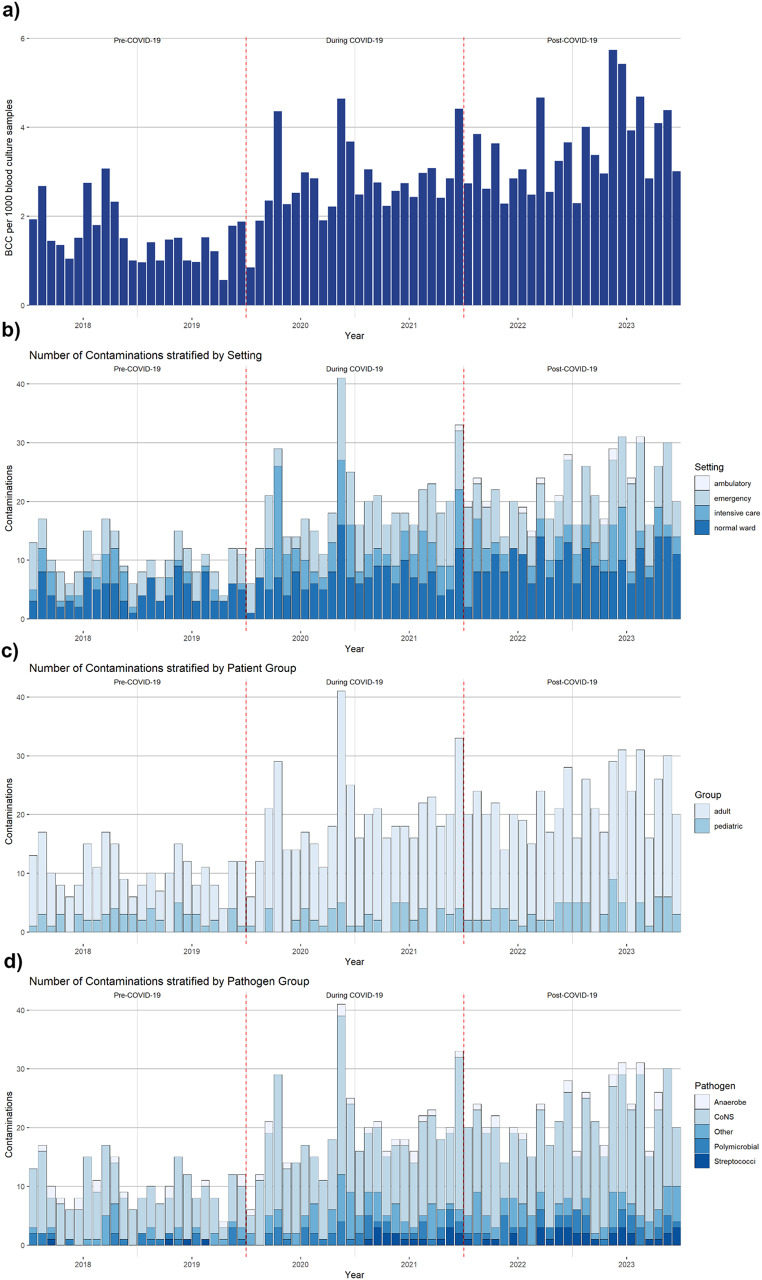
Monthly BCC incidence per 1000 blood culture samples (**a**) and BCC (absolute number) stratified by setting (**b**), patient (**c**), and pathogen group(**d**)

The monthly incidence of BCC increased during the study period (Fig. 1). The lowest monthly incidence was 0.56/1000 samples (October 2019), while the highest was 5.74/1000 samples (May 2023). The absolute numbers of BCC stratified for hospital departments, age group and causative organism is illustrated (Table 1). The proportion of BCC in intensive care unit increased during-COVID-19 period and decreased to baseline in the post-COVID-19 period.

Compared with the pre-COVID-19 period, IRRs for BCC steadily increased during COVID-19 (IRR 1.84, 95% CI 1.58–2.15, *p* < 0.001) and after COVID-19 peak (IRR 2.29, 95% CI 1.97–2.66, *p* < 0.001). After excluding BCCs due to contaminants classified in 2021, we observed the same trend during COVID-19 (IRR 1.84, 95% CI 1.57–2.14, *p* < 0.001) and after COVID-19 (IRR 2.12, 95% CI 1.81–2.48, *p* < 0.001). The BCC increase was also observed in a sensitivity analysis with a different period stratification into early COVID-19, late COVID-19 and post-COVID-19 peak periods (supplementary Table 2).

## Discussion

We conducted an observational study on BCC in a large hospital network revealing that the incidence of BCC increased during COVID-19 and remained elevated in the post pandemic period. Several studies reported an increase in BCC during COVID-19, yet all these studies were performed in the early pandemic phase and data from the post-pandemic period is scarce. Andrei et al. described an elevated BCC rate in 2020 compared to 2016 in a Swiss tertiary hospital [8]. Sacchetti et al. showed a significant rise in BCC rates across all non-emergency hospital departments during the COVID-19 pandemic [9]. However, Farfour et al., while reporting increased contamination rates during the first wave of COVID-19, observed a return to pre-COVID-19 levels during the second wave in two French hospital laboratories [6]. We observed a sustained increase in BCC rates in the period after the pandemic peak. In our study most BCCs were attributed to general wards and the number of BCC increased across the different populations suggesting that BCC remains a widespread and pervasive issue throughout the hospital network. The observed increase in observed BCCs in the ICU setting during COVID-19 might be attributable to an increased number of patients with SIRS in the ICU during that time, as well as overcrowding of the ICU, possibly leading to simple infection prevention measured not being strictly applied. Moreover, a program to reduce BCC was potentially re-implemented more effectively compared to other wards in the post-COVID-19 period. The rate of BCC occurring in the paediatric ward was least affected by the COVID-19 pandemic. This might be due to children being less likely to get infected or have severe symptoms of COVID-19, therefore limiting the impact on the respective wards [10].

Overall, BCC is an indicator of quality of care. It reflects inadequate aseptic practices such as poor collection technique and insufficient skin disinfection [11]. Risk of BCC may arise during any sampling procedure and is impacted by numerous factors, including availability of and adherence to standard operating procedures for sample collection, proper training of medical staff, working conditions, site of blood sample collection and patient related-factors [1]. The exact cause of the sustained increase in contamination rate after the COVID-19 period remains unresolved but might be attributed to factors such as stressful working conditions, unique work settings, staff turnover, collateral damage to medical education in the pandemic, and challenges posed by personal protective equipment, all of which may complicate the sample collection process. Furthermore, factors such as pandemic fatigue causing decreased adherence to hygiene protocols might play an important role [12]. We suggest conducting a qualitative study among healthcare professionals to evaluate blood culture collection approaches and identify potential factors contributing to the increase in BCC incidence. Based on the current analysis, we are starting an initiative to improve overall compliance with our local protocol, including adherence to infection prevention and control measures such as hand hygiene, appropriate glove handling and bottle and skin antisepsis. Notably, BCCs are often neglected in bloodstream infection surveillance, but it is important to acknowledge their clinical impact albeit not indicating a true infection.

Our study has several limitations. First, the results may not be generalizable to other centers due to variations in local protocols for blood culture sampling and hygiene practices. However, our hospital network includes multiple sites, which increases patient population diversity. Second, the direct relation to the COVID-19 pandemic cannot be established using an observational study design. However, the prospective investigation of BCCs across the study period increases study data quality. Third, the change in classification of contaminants in 2021 may have biased our results. Nevertheless, we performed a sensitivity analysis ruling this out. Due to limitations in our dataset, a stratified incidence analysis in the respective settings was not possible but might provide further insights in future analyses.

## Conclusions

We observed a substantial surge in BCC after the COVID-19 pandemic. The persistence of elevated BCC incidence in the post-pandemic period underscores the need for maintaining heightened awareness, periodic training, and rigorous standards of blood culture collection.

## Electronic supplementary material

Below is the link to the electronic supplementary material.

Supplementary Material 1

## Data Availability

The datasets generated and/or analysed during the current study are not publicly available due to patient confidentiality. Data may be made available on reasonable request, and these can be addressed to the corresponding author.
