# Supplementary material for: Increased incidence of blood culture contaminations during and after the COVID-19 pandemic
Source: Infection. 2025 Mar 3;53(2):711–6. doi: 10.1007/s15010-024-02469-6 (PMC11971130; doi:10.1007/s15010-024-02469-6)
Supplement: Supplementary file 1 — Supplementary Material 1 [file 15010_2024_2469_MOESM1_ESM.docx]

**Electronic Supplementary material**

**Supplementary methods:**

Blood culture prevention measures.

In general, two blood cultures (four bottles) are drawn if a bacterial infection is suspected. Three blood cultures are drawn if an intravascular infection is suspected. Each blood culture bottle is inoculated with 8-10 mL of blood. In patients with central venous catheters, blood cultures are drawn through both the catheter and a peripheral puncture. During the procedure non-sterile gloves are worn. The phlebotomy site is disinfected with 2% alcoholic chlorhexidine. Although blood culture bottles are covered with lids, the rubber septa of the vials are not sterile, and it is standard practice to disinfect the tops of culture bottles before inoculation. Sterile drapes are not routinely used. Procedures are standardized.

New Microorganisms defined as contaminants in 2021.

In 2021, some microorganisms were introduced as potential contaminants (common commensals) in our definition according to the CDC/NHSN list.

Before 2021, skin contaminants were: coagulase-negative staphylococci, *Micrococcus* spp., *Propionibacterium acnes*, *Bacillus* spp., *Corynebacterium* spp. The investigation could still conclude to a BCC even with micro-organisms other than skin contaminants.

The list of common commensals also includes (non-exhaustive) from 2021: viridans group streptococci, *Granulicatella adiacens, Aerococcus* spp, *Actinomyces* spp, *Gordonia* spp, *Dermobacter hominis*

**Supplementary results**

Table 1: Distribution of streptococcal species during the three study periods

| **Group** | **Genus** | **Species** | **Pre- COVID-19** | **During COVID-19** | **Post-**  **COVID-19 peak** |
| --- | --- | --- | --- | --- | --- |
| Viridans alpha-haemolytic | *Streptococcus* | *mitis; oralis ; anginosus; cristatus; constellatusi; gordonii; intermedius ; milleri; parasanguinis; salivarius; sanguinis* | 4 | 17 | 32 |
| Beta-haemolytic group a | *Streptococcus* | *pyogenes* | 0 | 0 | 0 |
| Beta-haemolytic | *Streptococcus* | *agalactiae* | 0 | 0 | 1 |
| Alpha-haemolytic group c or g | *Streptococcus* | *canis; dysagalactiae; equisimilis; peroris; pseudopneumoniae; urinalis* | 0 | 0 | 0 |
|  |  | *alactolyticus; bovis; criceti; equinus; gallolyticus; hyovaginalis; infantriusa; infanti; lutetiensis; macacae; mutans; ratti; sinensis; sobrinus; trigurinus* | 0 | 0 | 1 |

Table 2: Sensitivity analyses with additional stratification of COVID-19 periods

| Incidence rate ratios | IRR | 95% CI | p-value |
| --- | --- | --- | --- |
| Contaminations (reference=pre COVID-19 years 2018-2019) | | | |
| First COVID-19 period (2020) | 1.81 | 1.51-2.17 | <.0001 |
| Second COVID-19 period (2021) | 1.87 | 1.57-2.23 | <.0001 |
| Post COVID-19 peak (2022-2023) | 2.29 | 1.97-2.66 | <.0001 |
| Contaminations without considering new microorganisms (reference=pre COVID-19 years 2018-2019) | | | |
| First COVID-19 period (2020) | 1.88 | 1.57-2.17 | <.0001 |
| Second COVID-19 period (2021) | 1.79 | 1.49-2.23 | <.0001 |
| Post COVID-19 peak (2022-2023) | 2.12 | 1.81-2.66 | <.0001 |

Legend. IRR: Incidence rate ratio. CI: Confidence interval.
